# A review of functional slit lamp biomicroscopy

**DOI:** 10.1186/s40662-019-0140-7

**Published:** 2019-05-21

**Authors:** Xupeng Shu, Jianhua Wang, Liang Hu

**Affiliations:** 1 School of Ophthalmology and Optometry, Wenzhou Medical University, Wenzhou, 325027 China; 2 Bascom Palmer Eye Institute, University of Miami, Miami, Florida, USA

**Keywords:** Functional slit lamp biomicroscopy (FSLB), Conjunctival blood flow velocity, Microvascular network density, Contact lens, Dry eye disease

## Abstract

Functional slit lamp biomicroscopy (FSLB) is a novel device which consists of a traditional slit-lamp and a digital camera. It can quantitatively assess vessel diameter, blood flow velocity, and blood flow rate and can create noninvasive microvascular perfusion maps (nMPMs). At present, FSLB is mainly used in contact lens (CL) and dry eye disease (DED) studies to advance our understanding of ocular surface microcirculation. FSLB-derived blood flow and vessel density measures are significantly altered in CL wearers and DED patients compared to normal people. These subtle changes in the ocular surface microcirculation may contribute to the monitoring of potential diseases of the body and provide a new way to diagnose dry eye disease. Therefore, this may also indicate that FSLB can be more widely applied in the study of other diseases to reveal the relationship between changes in ocular surface microcirculation and systemic diseases. The purpose of this paper is to summarize the functions of FSLB and the related studies especially in CL and DED.

## Background

The conjunctiva is a translucent mucosal tissue attached to the sclera that contains a large number of vascular networks [1], and is directly exposed to the environment. This feature allows real-time, in vivo, noninvasive detection of the bulbar conjunctival microvasculature. Moreover, changes in the microvasculature may indicate the occurrence and progression of some systemic diseases. Some researchers have discovered a potential relationship between conjunctival microvasculature and ocular, cerebral, and systemic diseases such as dry eye syndrome [2], sickle cell anemia [3, 4], diabetes [5–7], and Alzheimer’s disease [8, 9] through qualitative assessment [3, 5, 6, 8, 10–12]. To better detect the conjunctival microvasculature, several techniques have been developed to examine the conjunctival microvasculature in vivo, including slit-lamp stereomicroscopy, laser Doppler flowmetry, orthogonal polarized spectral imaging, modified scanning laser ophthalmoscopy, adapted slit-lamp biomicroscopy digital imaging and computer-assisted intravital microscopy (CAIM) [4, 5, 7, 10, 13–20]. However, none of these systems could image both the small and large fields or create noninvasive microvascular perfusion maps (nMPMs). Compared with the previous methods, functional slit-lamp biomicroscopy (FSLB) can quantitatively assess vessel diameter, blood flow rate and velocity as well as create nMPMs. This technique may be more sensitive than other methods for detecting earlier vasculopathic changes. At present, FSLB has been applied in the fields of contact lens (CL) and dry eye disease (DED) studies and this paper will review the functions of FSLB and the related studies especially in CL and DED.

## Main text

### Functional slit-lamp biomicroscopy

**Fig. 1:**
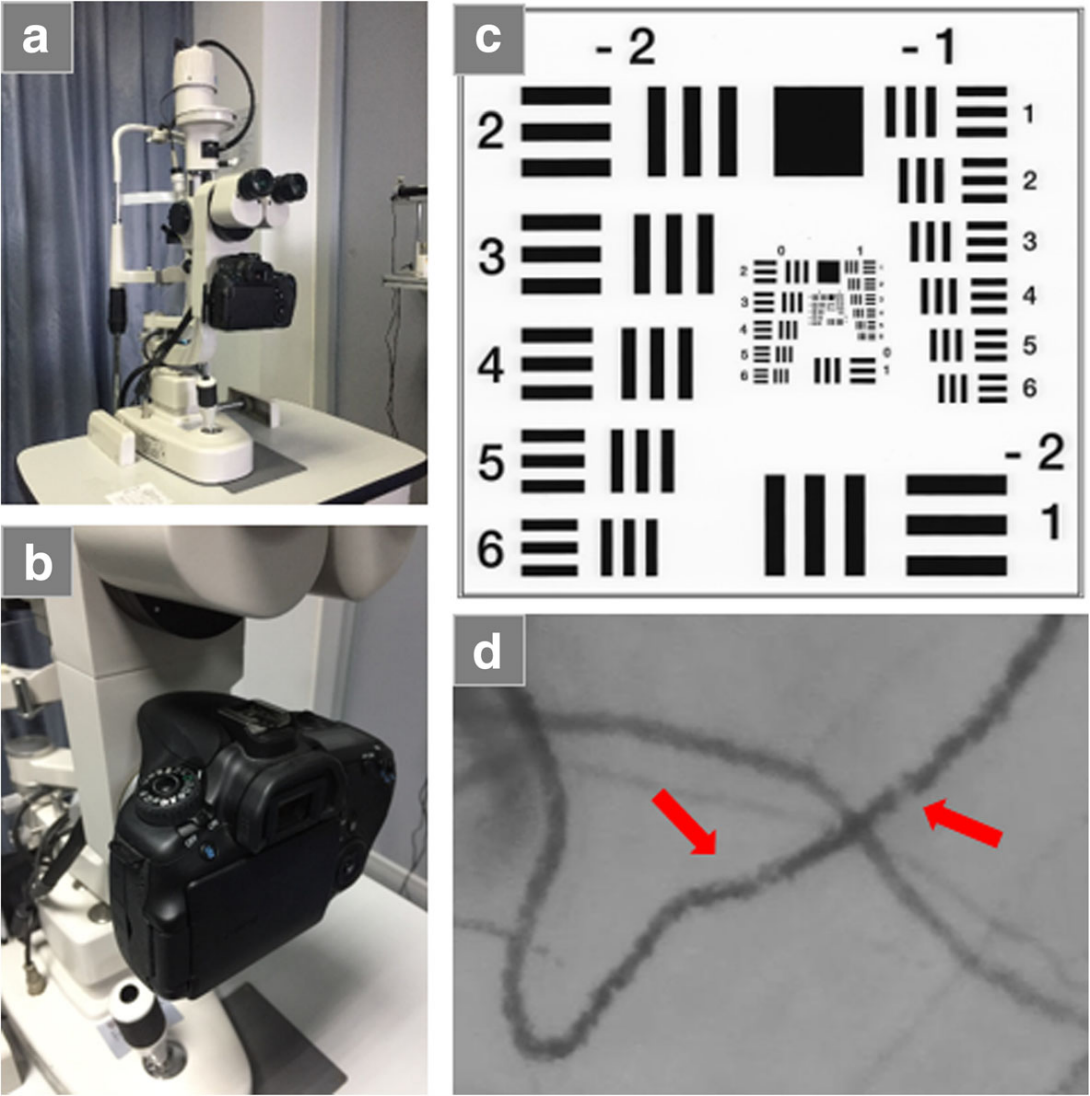
Functional slit-lamp biomicroscopy. The functional slit-lamp biomicroscopy (FSLB) is a combination of a high-speed digital camera (Canon 60D) and a traditional slit lamp (**a**, **b**). The spatial display resolution is tested using a resolution test card (US Air Force 1951, Edmund Optics Inc., Barrington, NJ) (**c**), and the resolution of the FSLB is 3.11 μm. With the inherent optical magnification of 30× and additional 7× magnification provided by the Movie Crop Function, the total magnification is 210×, which is sufficient to image the movement of red blood cell aggregates. Clusters of red blood cells (red arrows) are clearly visible (**d**)

The FSLB imaging system consists of a traditional slit-lamp and a digital camera (Canon 60D, Canon Inc., Melville, NY)(Fig. 1a and b). The camera has a Movie Crop Function, which enables about 7× magnification without the loss of image quality for high speed video recording at 60 fps. With built-in optical magnifications of up to 30× (set by a dial on the slit-lamp), total magnification can reach approximately 70 to 210×. When the total magnification with the Movie Crop Function is set up to ~ 210×, the image resolution can reach 3.11 μm (tested by US Air Force 1951 resolution test card)(Fig. 1c). The setup is sufficient to image the red blood cell (RBC) aggregate movement (Fig. 1d) for the measurement of blood flow velocity, flow rate and vessel diameter [1, 21]. In addition, the system can create nMPMs. FSLB has been used to image the microvascular network of the bulbar conjunctiva, upper tarsal conjunctiva or lid wiper [22], and fractal analysis was performed to determine fractal dimensions. The box-counting analysis method was used to obtain Dbox (representing vessel density), and the fractal dimension from multifractal analysis yielded D0 (representing vessel complexity) [21, 23].

## Function

### Imaging bulbar conjunctival hemodynamics

**Fig. 2:**
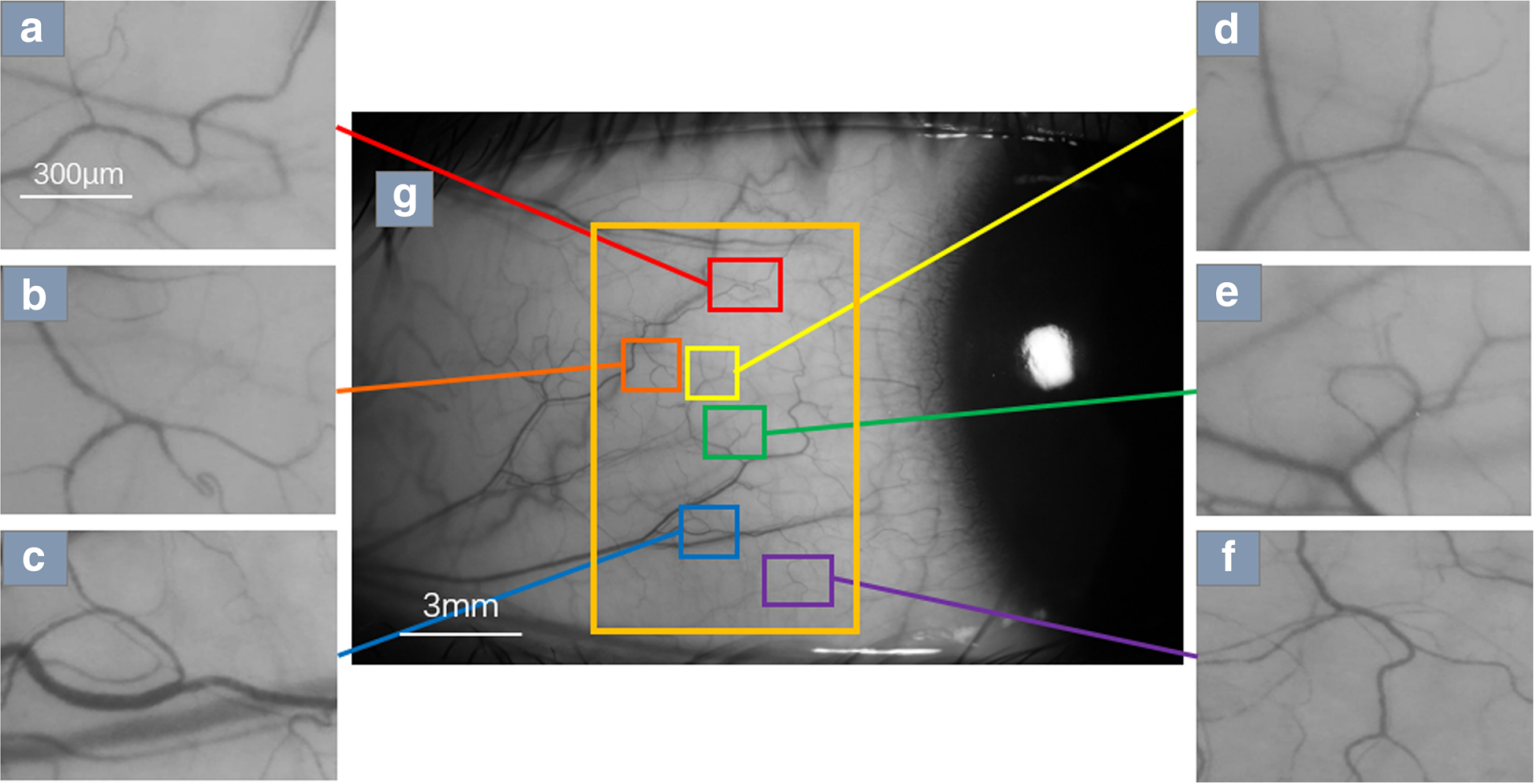
The location and imaging effect of functional slit lamp biomicroscopy. Temporal bulbar conjunctival microvasculature imaged by FSLB. Video clips are acquired at 6 locations, which are homogeneously overlaid on the temporal bulbar conjunctiva (**g)**. With a magnification of 175×, red blood cell clusters can be seen in all 6 fields (**a** to **f**)

To capture the movement of RBC aggregates clearly, the total magnification of the FSLB can be set up to ~ 210× (or ~ 175×); the system has 1.46 μm per pixel at the highest magnification (~ 210×) for a field of view of 0.94 × 0.70 mm^2^, and it has 1.902 μm per pixel at the lower magnification (~ 175×) for a field of view of 1.22 × 0.91 mm^2^; for the two different magnification settings, an image size of 640 × 480 pixels was utilized [21, 24, 25]. A typical magnification for reliable measurement of RBC velocity must provide at least one pixel and at best a few pixels for one RBC (~ 7 μm) to obtain a good signal-to-noise ratio [26]. The two different settings provide approximately 3.7–5 pixels for one RBC, and the FSLB system has 60 fps, which is sufficient to capture RBC velocities of up to 2.6 mm/s. When the magnification is adjusted to 175–210×, the flow of red blood cells can be clearly captured. The red blood cell flow video captured in the Movie Crop Function mode can be used to calculate parameters such as microvascular diameter, blood flow velocity and blood flow rate through a series of video image processing programs in the custom software based on MATLAB. Figure 2 shows the flow of red blood cells taken at 175× magnification.

### Process method

**Fig. 3:**
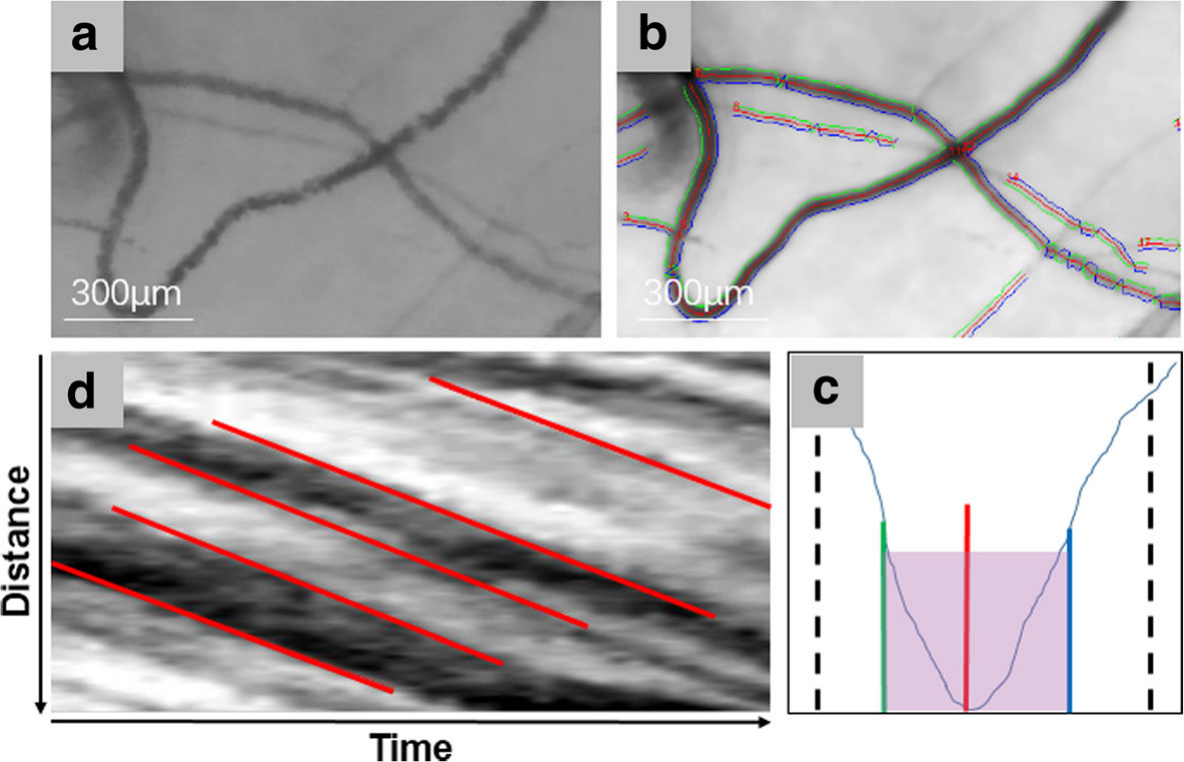
Analyzing hemodynamics and vessel diameter. The custom software based on MATLAB can compensate for the rotation of the eye in the captured video and can convert the dynamic video into a picture (**a**). The software will automatically identify the vascular signal in the picture and will mark the contour of the blood vessel (**b**). Vessels are segmented from the averaged image of all frames in the video, and centerlines of the vessels are marked in red; then, the software searched for the centerline of 75 pixels and created intensity profiles, which are perpendicular to the centerline. The vessel diameter is defined as the full width at the half-maximum (FWHM) of the intensity profile, and the vessel wall is marked in green and blue lines (**c**). The blood flow velocity can be calculated through the generation of space-time images (**d**); the abscissa represents time, the ordinate represents the distance traveled by the blood flow signal, the red line segment is drawn according to the blood flow signal, and the slope of the line segment represents the blood flow velocity

This process is shown in Fig. 3. The first step is to capture bloodstream dynamic video and convert it into a series of images by custom software based on MATLAB. To ensure that more accurate results are calculated, at least half a second of video is collected for each image area and converted to at least 30 consecutive frames. The first frame image is used as a reference frame for compensating for eye movement. To measure blood flow velocity, a space-time image (STI) is created, with the y-axis representing the length of the vessel path and the x-axis being the time in the video. The red line segment is drawn according to the blood flow signal, and the slope of the red line segment is calculated as the measured value of the axial blood flow velocity using the linear equation y = mx + b (where m determines the slope and b is the intercept). The cross-sectional blood flow velocity and blood flow rate can be calculated using equations by Koutsiaris [27]. The average diameter of the blood vessels is calculated based on the image converted from the video clip of the measured speed. The vessel diameter is defined as the full width at the half maximum (FWHM) of the intensity distribution, which is marked with green and blue lines [21, 24]. More detailed processing is documented in Jiang’s paper [21].

### Imaging bulbar conjunctival nMPMs and fractal analysis

Fractal analysis is a tool commonly used to analyze blood vessel branch networks in fundus images, and vasculopathies represented by fractal dimensional changes have been identified in stroke, diabetic retinopathy and ocular conditions [28–30]. Schulze et al. found that fractal analysis also can be used to observe and assess conjunctival redness/congestion [31]. For imaging the microvascular network, a built-in green filter is used with a setting of 22× magnification within the slit-lamp (or 16× magnification, depending on the model of the slit lamp) to obtain a field of view of approximately 15.74 × 10.50 mm^2^ (approximately 14.63 × 9.75 mm^2^ with the setting of 16× magnification), a diffuse filter is used to take the high pixel image (5184 × 3456 pixels) of the temporal bulbar conjunctiva. The vascular network image will then be output and processed through a series of image processing programs and fractal analysis will be performed by the box counting method. The mono-fractal (Dbox) and multi-fractal (D0) values are obtained to evaluate vessel density and complexity of the nMPMs [18, 21, 24, 25].

### Process method

**Fig. 4:**
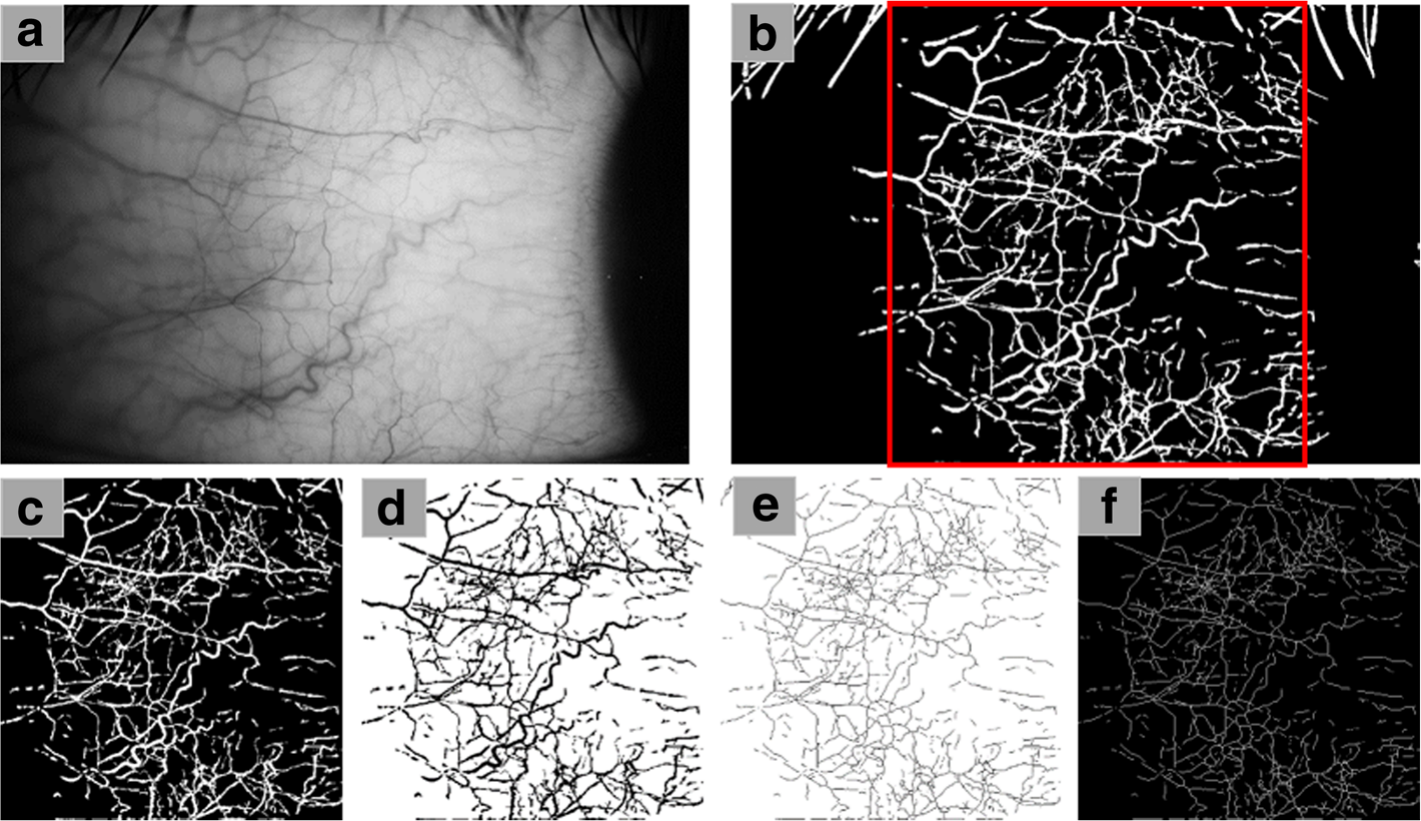
Analyzing Dbox and D0. The original image was converted to grayscale to reduce image resolution (**a**). Noninvasive microvascular perfusion maps (nMPMs) (**b**) were generated by custom software, and vascular image of 512 × 512 pixels (marked as red square) was intercepted for skeletonization (**c** to **f**). The image (**f**) can be analyzed by Mono-fractal and Multi-fractal analysis to obtain the measurements for Dbox and D0

This process is shown in Fig. 4. The original image of the conjunctiva needs to be grayed out to reduce the image resolution and this process will adjust the image from 5184 × 3456 pixels to 1024 × 683 pixels (Fig. 4a). The image is then processed using custom software based on MATLAB, which is fully automated and removes nonvascular structures from the image to create a vascular network image (Fig. 4b). ImageJ (NIH, Bethesda, MD) is used to crop the image to 512 × 512 pixels that covers 7.87 × 7.87 mm^2^ near the edge (Fig. 4c) and then invert it. After making the binary of the image (Fig. 4d), the image is hollowed out (Fig. 4e) and then inverted to be ready for fractal analysis (Fig. 4f). The fractal dimension of the segmented bulbar conjunctiva nMPM is analyzed using a fractal analysis toolbox from BenoitTM (TruSoft Benoit Pro 2.0, TruSoft Inc., St. Petersburg, FL). Mono-fractal (Dbox) and multi-fractal (D0) are both analyzed. More detailed processing methodology is documented in Jiang’s paper [21].

## Research application

### Healthy eye

The conjunctival microvasculature is directly exposed, and the microcirculation parameters of the bulbar conjunctiva may be altered by external environmental factors, which will cause great interference for future research. Therefore, it is important to understand whether the microcirculation parameters of the bulbar conjunctiva are stable in daily activities. Xu et al [18] observed that there were no significant variations in the vessel diameter and hemodynamics in healthy people during office hours (from 9 AM to 5 PM, as most clinical studies and patient care are performed during this time), whereas the fractal dimensions of the nMPMs were significantly increased at 3 PM and 5 PM compared with the baseline obtained at 9 AM [18]; results are shown in Table 1. This finding is similar to that of Duench’s study, and it may indicate that fractal analysis has higher sensitivity compared with other measurement parameters in the application parameters of FSLB [14, 18]. At the same time, the repeatability of FSLB was also verified in this study, the results showed that for measuring the vessel diameter between two graders, CoR% (the percentage of coefficient of repeatability) and ICC (intraclass, interclass and intersession correlation coefficient) values were 4.87% and 0.989, respectively, and for axial blood velocity, the CoR% and ICC were 11.49% and 0.997, respectively [18]. The repeatability of FSLB can be considered good [18]. In addition, the accuracy of FSLB in measuring blood flow velocity was close to 100% by comparing with the known velocity [21].

**Table 1:** Summary of significant functional slit-lamp biomicroscopy findings on the ocular surface

Reference	*N* (eyes)	Group	Blood flow velocity (mm/s)	Blood flow rate (pl/s)	Vessel diameter (μm)	Dbox	D0
Xu et al. [18]	20	9 am vs. 5 pm	0.62 ± 0.31 (9 am) 0.63 ± 0.25 (5 pm)	166.20 ± 85.78 (9 am) 158.03 ± 69.57 (5 pm)	21.68 ± 2.47 (9 am) 21.05 ± 2.79 (5 pm)	**1.61** ± **0.06** (**9 am**) **1.64** ± **0.04** (**5 pm**) (*)	**1.68** ± **0.04** (**9 am**) **1.70** ± **0.04** (**5 pm**) (*)
Jiang et al. [21]	6	Baseline vs. 6h after CL wear	**0.60 ± 0.12** (**B**) **0.88 ± 0.21** (**6 h**) (*)	**129.8 ± 59.9** (**B**) **207.2 ± 81.3** (**6h**) (*)	**18.8 ± 2.7** (**B**) **19.6 ± 2.4** (**6 h**) (*)	**1.63** ± **0.05** (**B**) **1.69** ± **0.03** (**6 h**) (*)	**1.71** ± **0.03** (**B**) **1.76** ± **0.03** (**6 h**) (*)
Chen et al. [32]	22	Baseline vs. 6h after CL wear	**0.51** ± **0.20** (**B**) **0.65** ± **0.22** (**6 h**) (*)		17.8 ± 1.8 (B) 17.9 ± 1.2 (6 h)		
Shi et al. [23]	60	HCL vs. NCL	**0.59** ± **0.19** (**H**) **0.48** ± **0.17** (**N**) (*)	**119** ± **38** (**H**) **92** ± **39** (**N**) (*)	16.9 ± 1.3 (H) 17.3 ± 1.6 (N)	**1.64** ± **0.05** (**H**) **1.61** ± **0.05** (**N**) (*)	**1.71** ± **0.05** (**H**) **1.69** ± **0.04** (**N**) (*)
Hu et al. [33]	166	HCL vs. NCL Female vs. Male	**0.61 ± 0.15** (**H**) **0.50 ± 0.14** (**N**) (*) **0.51 ± 0.14** (**F**) **0.42 ± 0.13** (**M**) (*)	**140** (**H**) **94.5** (**N**) (*) **93.7** (**F**) **80.4** (**M**) (*)	**18.3** (**H**) **16.4** (**N**) (*) 16.2 (F) 16.6 (M)	**1.67** (**H**) **1.62** (**N**) (*) 1.61 (F) 1.62(M)	**1.75** (**H**) **1.69** (**N**) (*) 1.68 (F) 1.69 (M)
Deng et al. [22]	16	Tarsal conjunctiva (T) vs. Lid wiper (L) vs.Bulbar conjunctiva (B)				**1.731** ± **0.026** **1.740** ± **0.030** (T*) **1.411** ± **0.116** **1.548** ± **0.079** (L*) **1.587** ± **0.059** **1.632** ± **0.060** (B*)	
Chen et al. [24]	56	Baseline vs. After an air puff	**0.50 ± 0.15** (**B**) **0.55** ± **0.17** (**A**) (*)		22.13 ± 1.84 (B) 22.21 ± 2.04 (A)	1.64 ± 0.04 (B) 1.65 ± 0.04 (A)	
Chen et al. [25]	50	DED patients vs. Healthy human	**0.59 ± 0.09** (**D**) **0.47 ± 0.12** (**H**) (*)	**169.5 ± 1.8** (**D**) **107.2 ± 49.6** (**H**) (*)	**21.8 ± 1.8** (**D**) **17.9 ± 2.2** (**H**) (*)	**1.65 ± 0.04** (**D**) **1.60 ± 0.07** (**H**) (*)	

*CL* = contact lens; *HCL* = Habitual contact lens; *NCL* = Noncontact lens; *DED* = Dry eye disease

(*), ***P*** **<0.05**

Bold fonts indicate statistical differences in data comparison

The conjunctiva has a large number of blood vessels. To improve the reliability of the study, it is important to determine blood vessel sampling size. According to Wang’s study [1], approximately 15 blood vessels can represent the overall blood vessel diameter and blood flow velocity distribution, and the diameter and velocity distributions were not only unimodal but were also somewhat positively skewed and abnormal in healthy people [1]. In addition, studies have shown that the blood flow velocity of bulbar conjunctiva was positively correlated with vessel diameter [1, 32]. Higher blood flow velocity and blood flow rate of the bulbar conjunctiva were also observed in females compared with males [33].

These findings suggest that FSLB can quantitatively measure conjunctival hemodynamics and morphological parameters and laid the foundation for future research on conjunctival microcirculation.

### Short-term soft contact lens wearer

After wearing soft contact lenses (CL) for a short time, the bulbar conjunctiva microcirculation will change significantly [21, 32]. Jiang et al [21] found that the morphometry and hemodynamics of the bulbar conjunctival microvasculature and the fractality of the bulbar conjunctival microvascular network were significantly increased after wearing CL for 6 h (Table 1). Similarly, Chen et al [32] also found that the blood flow velocity of the bulbar conjunctival microvasculature was significantly increased after wearing CL for 6 h, and the increased velocities were found across all the vessel diameter ranges (Table 1). In addition, blood flow velocity and blood flow rate were both positively correlated with vessel diameter at baseline and after wearing CL for 6 h. These may indicate that the increased blood flow velocity measured in the bulbar conjunctiva outside the area underneath the lens edge may be a response to the compressed vessels, which results in decreased blood supply to the cornea [32]. Furthermore, Deng et al [22] found that after 6 h of CL wear, the microvascular network densities were increased in the upper tarsal conjunctiva, lid wiper, and bulbar conjunctiva (Table 1) and that the decrease in ocular discomfort was strongly related to the density change in the lid wiper [22]. These findings can inspire the execution of future studies on monitoring the conjunctival microvasculature by FSLB for lens design or for health management of the ocular surface.

### Long-term soft contact lens wearer

Significant changes in the bulbar conjunctiva microcirculation can occur after wearing soft CL for a short period of time. Similarly, changes have been found among people who have worn soft CL for a long time(at least 6 months). Shi et al [23] used FSLB to examined conjunctival microvasculature development in long-term habitual contact lens (HCL) wearers. FSLB was used to image the temporal bulbar conjunctiva in the morning while the CL wearers were not wearing their lenses after a night of sleep. The results showed that the average blood flow velocity and the microvascular network density and complexity levels in HCL wearers after a night of sleep were significantly higher than that in noncontact lens (NCL) wearers (Table 1). Moreover, the blood flow velocity was positively correlated with the duration of CL wear (r = 0.46, P<0.05) and with the daily number of lens-wearing hours (r = 0.49, P<0.05) in HCL wearers [23]. This finding suggests that vascular alterations, such as increased blood flow velocities and microvascular networks, do not recover after a night of sleep without wearing lenses and suggests a cumulative effect that maintains chronic inflammation in HCL wearers. Similarly, Hu et al [33] found that the blood flow velocity, vessel diameter, microvascular network density and complexity levels of HCL wearers were significantly higher than those of NCL wearers (Table 1). In addition, they found that the blood flow velocity was positively correlated with CL hours of wear per day and with CL days of wear per week in HCL wearers, and it was negatively correlated with age in the NCL wearers. These findings can be explained by the occurrence of para-inflammation or subclinical chronic inflammation, which is considered as the middle ground between the basal state and infected or damaged states [23, 33] owing to elevated immune activity, creating a steady state of alertness. Briefly, wearing CLs triggers or modulates vascular responses, which are maintained at a low level between basal and extreme states, and this mechanism may serve as a protective function to protect the CL wearer from serious complications such as corneal infection, corneal ulcers, and keratitis. This hypothesis can be supported by the Manchester Keratitis study [34–36], which showed that the protective mechanism could be mediated by recruitment of macrophages and Langerhans cells [37, 38].

**Fig. 5:**
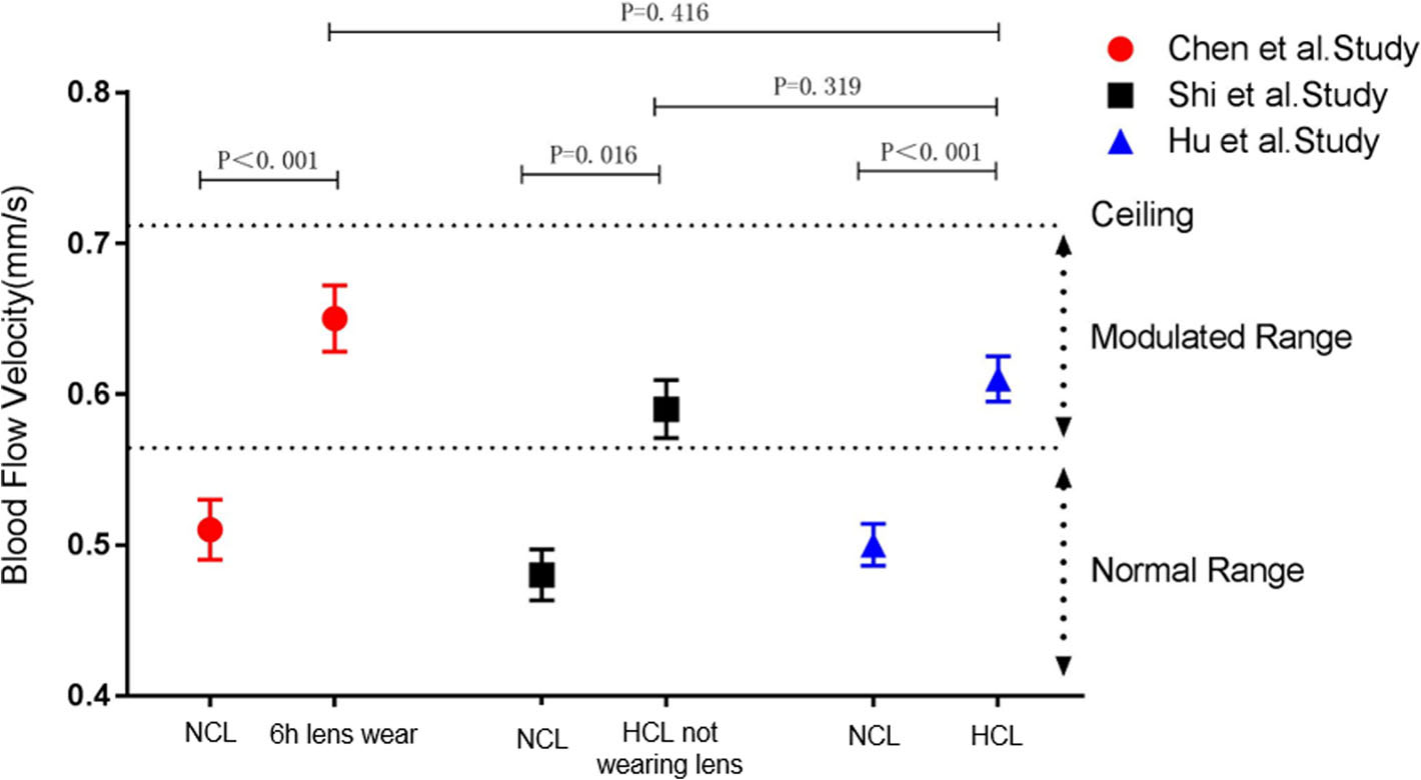
Comparison of blood flow velocity (BFV) among soft contact lens (CL) studies using the same imaging protocol. Chen et al. examined the BFVs of 22 NCL wearers after 6 h of CL wear and found a significant increase in BFVs. Shi et al. examined 20 HCL wearers who were imaged when they were not wearing their CLs after one night of sleep and found that BFV remained higher compared with that of 40 NCL wearers. In the Hu et al. study, a significantly higher BFV was observed in 75 HCL wearers than in 91 NCL wearers. Comparing BFVs of HCL wearers in the Hu et al. study with those of NCLs after 6 h of CL wear and with those of HCLs when they were not wearing their CLs, no significant differences were found. Overall, it appeared that wearing lenses caused an elevated BFV, and a possible ceiling effect limited the evaluation within a modulated range [33]

**Fig. 6:**
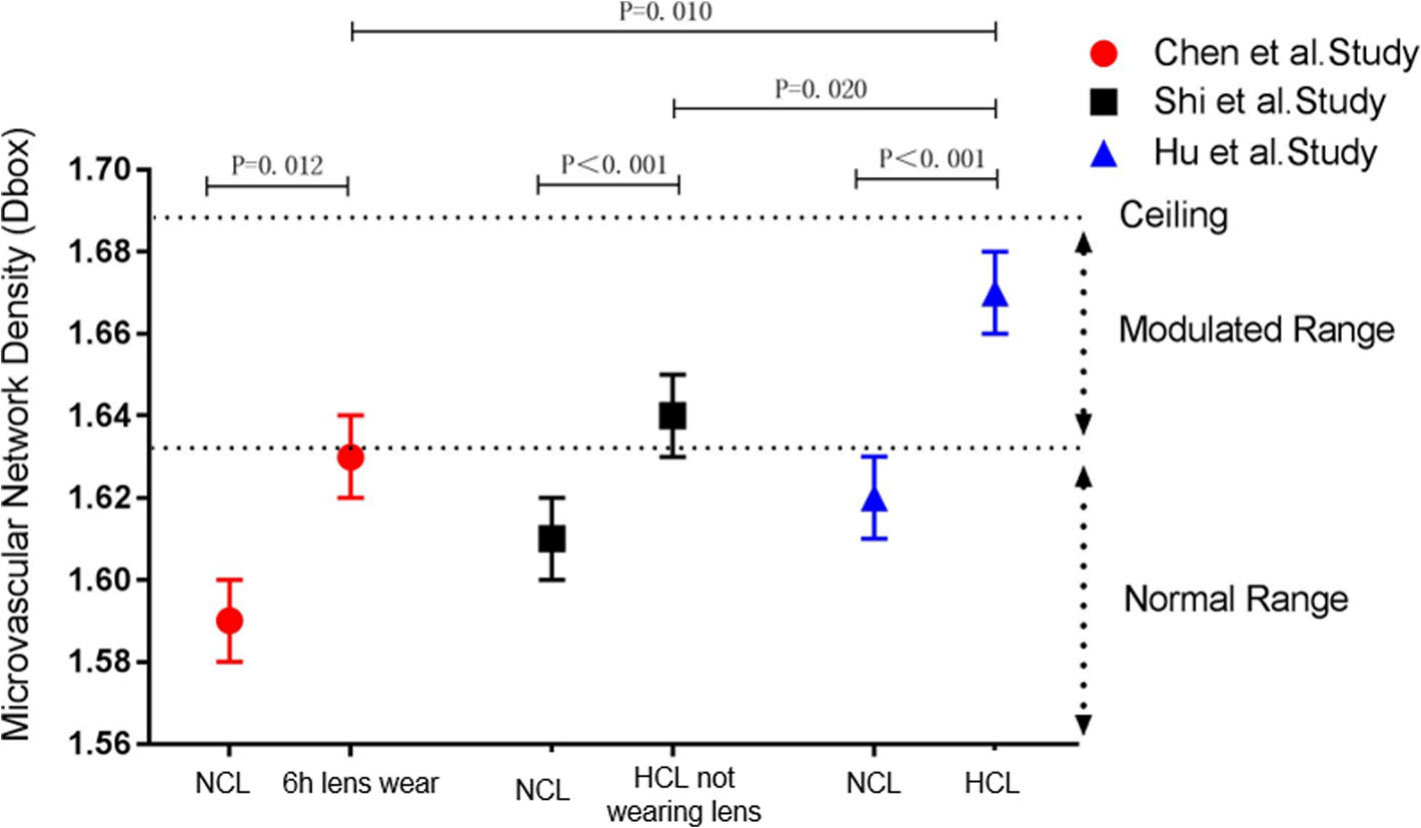
Comparison of microvascular network density (Dbox) among soft contact lens (CL) studies using the same imaging protocol. Chen et al. examined 16 NCL wearers and found a significantly elevated Dbox after 6 h of CL wear. Shi et al. examined 20 HCL wearers who removed their CLs for one night of sleep and found an elevated Dbox compared with 40 NCL wearers. In the Hu et al. study, a significantly higher Dbox was identified in 75 HCL wearers compared with 91 NCL wearers. The Dbox of HCL wearers while wearing CLs was significantly different from that when they were not wearing CLs and from that of NCL wearers after 6 h of CL wear. Overall, it appeared that wearing lenses caused elevated vessel density and a possible ceiling effect limited the evaluation within the modulated range [33]

Figures 5 and 6, which were cited from Hu’s paper, summarize and compare relevant studies on contact lens use.

### Dry eye disease

Dry Eye Disease (DED) is a heterogeneous disease of the ocular surface, including but not limited to the lacrimal functional unit (LFU). It is characterized by symptoms of various intensity that may include ocular pain (described as dryness, burning, and aching) and visual complaints (fluctuating or blurry vision), both of which can reduce quality of life [39, 40]. Chen et al [24] found that after central corneal stimulation with an air puff in DED patients, bulbar blood flow velocity was increased (Table 1) and those with higher self-rated wind hyperalgesia demonstrated less change in blood flow velocity after air stimulation on the central cornea. Moreover, baseline blood flow velocity was positively associated with Schirmer scores (r = 0.40, *P* = 0.002) in DED patients, indicating that mechanical stimuli on the central cornea affect overall bulbar conjunctiva blood flow velocity and differentially in patients with neuropathic ocular pain (NOP) complaints [24]. In addition, DED patients might be a heterogeneous group with different functions in the corneal somatosensory pathway, efferent responses to sensory stimuli, and autonomic nervous states [24]. These findings may indicate that assessment of the conjunctival microvascular parameters can help diagnose the DED subtype, guide treatment, and follow treatment response. In fact, Chen et al [25] observed more microvascular alterations in the bulbar conjunctiva of DED patients than in healthy people, including increased blood flow velocity and blood flow rate, higher vessel density and larger vessel diameter (Table 1). According to the ROC (Receiver Operating Characteristic) curves of blood flow velocity, vessel density and vessel diameter based on the OSDI (Ocular Surface Disease Index) standard, the results showed that the sensitivity and specificity of vascular measurements are highly compatible with those found in OSDI [25]. The above findings indicate direct evidence of the role of chronic inflammation in microcirculation and microvascular changes in dry eye patients. In addition, it may indicate that the use of FSLB for conjunctival microvascular assessment can provide a new method for DED diagnosis.

## Conclusions

Detection of functional parameter abnormalities in the conjunctiva may indicate the occurrence and progression of ocular disease such as DED. Furthermore, the characteristics of the conjunctiva allow real-time, in vivo, and noninvasive monitoring. FSLB is a novel imaging device for detecting conjunctival microvasculature; it consists of a traditional slit-lamp and a digital camera. FSLB can analyze the morphology and hemodynamic parameters of the conjunctival microvasculature, including vessel diameter, vessel length, blood flow velocity and blood flow rate. Moreover, it can create nMPM, and custom software can be used to analyze vessel density and complexity of the conjunctival microvascular network. In addition, the accuracy and repeatability of the FSLB was also demonstrated in previous studies. Currently, FSLB is mainly used in CL and DED studies. The advantages of the FSLB imaging system include being noninvasive and inexpensive, which allow the widespread use and easy implementation of the device in other studies or clinical diagnosis and treatment for DED and even systemic disease in the future. In short, FSLB has a broad application prospect.

## References

[1] Wang L, Yuan J, Jiang H, Yan W, Cintrón-Colón HR, Perez VL (2016). Vessel sampling and blood flow velocity distribution with vessel diameter for characterizing the human bulbar conjunctival microvasculature. Eye Contact Lens.

[2] Rodriguez JD, Johnston PR, Ousler GW, Smith LM, Abelson MB (2013). Automated grading system for evaluation of ocular redness associated with dry eye. Clin Ophthalmol.

[3] Cheung AT, Miller JW, Craig SM, Lin X, Samarron SL, To PL (2010). Comparison of real-time microvascular abnormalities in pediatric and adult sickle cell anemia patients. Am J Hematol.

[4] Wanek J, Gaynes B, Lim JI, Molokie R, Shahidi M (2013). Human bulbar conjunctival hemodynamics in hemoglobin SS and SC disease. Am J Hematol.

[5] Cheung AT, Tomic MM, Chen PC, Miguelino E, Li CS, Devaraj S. Correlation of microvascular abnormalities and endothelial dysfunction in type-1 diabetes mellitus (T1DM): a real-time intravital microscopy study. Clin Hemorheol Microcirc. 2009;42(4):285–95. 10.3233/CH-2009-1199 19628894

[6] Devaraj S, Cheung AT, Jialal I, Griffen SC, Nguyen D, Glaser N (2007). Evidence of increased inflammation and microcirculatory abnormalities in patients with type 1 diabetes and their role in microvascular complications. Diabetes..

[7] To WJ, Telander DG, Lloyd ME, Chen PC, Cheung AT. Correlation of conjunctival microangiopathy with retinopathy in type-2 diabetes mellitus (T2DM) patients. Clin Hemorheol Microcirc. 2011;47(2):131–41. 10.3233/CH-2010-1374 21339633

[8] Chew SH, Meighan Smith Tomic M, Cheung AT (2010). Alzheimer's disease: more than amyloid. Clin Hemorheol Microcirc.

[9] Smith MM, Chen PC, Li CS, Ramanujam S, Cheung AT (2009). Whole blood viscosity and microvascular abnormalities in Alzheimer's disease. Clin Hemorheol Microcirc.

[10] Cheung AT, Hu BS, Wong SA, Chow J, Chan MS, To WJ (2012). Microvascular abnormalities in the bulbar conjunctiva of contact lens users. Clin Hemorheol Microcirc.

[11] Cheung AT, Ramanujam S, Greer DA, Kumagai LF, Aoki TT (2001). Microvascular abnormalities in the bulbar conjunctiva of patients with type 2 diabetes mellitus. Endocr Pract.

[12] Cheung AT, Miller JW, Miguelino MG, Li J, Lin X, To WJ (2012). Exchange transfusion therapy and its effects on real-time microcirculation in pediatric sickle cell anemia patients: an intravital microscopy study. J Pediatr Hematol Oncol.

[13] Jung F, Körber N, Kiesewetter H, Prünte C, Wolf S, Reim M (1983). Measuring the microcirculation in the human conjunctiva bulbi under normal and hyperperfusion conditions. Graefes Arch Clin Exp Ophthalmol.

[14] Duench S, Simpson T, Jones LW, Flanagan JG, Fonn D (2007). Assessment of variation in bulbar conjunctival redness, temperature, and blood flow. Optom Vis Sci.

[15] Shahidi M, Wanek J, Gaynes B, Wu T (2010). Quantitative assessment of conjunctival microvascular circulation of the human eye. Microvasc Res.

[16] Koutsiaris AG, Tachmitzi SV, Papavasileiou P, Batis N, Kotoula MG, Giannoukas AD (2010). Blood velocity pulse quantification in the human conjunctival pre-capillary arterioles. Microvasc Res.

[17] Cheung AT, Harmatz P, Wun T, Chen PC, Larkin EC, Adams RJ (2001). Correlation of abnormal intracranial vessel velocity, measured by transcranial Doppler ultrasonography, with abnormal conjunctival vessel velocity, measured by computer-assisted intravital microscopy, in sickle cell disease. Blood..

[18] Xu Z, Jiang H, Tao A, Wu S, Yan W, Yuan J (2015). Measurement variability of the bulbar conjunctival microvasculature in healthy subjects using functional slit lamp biomicroscopy (FSLB). Microvasc Res.

[19] Ohtani N (1996). Laser Doppler flowmetry of the bulbar conjunctiva as a monitor of the cerebral blood flow. Nihon Kyobu Geka Gakkai Zasshi.

[20] Schaser KD, Settmacher U, Puhl G, Zhang L, Mittlmeier T, Stover JF (2003). Noninvasive analysis of conjunctival microcirculation during carotid artery surgery reveals microvascular evidence of collateral compensation and stenosis-dependent adaptation. J Vasc Surg.

[21] Jiang H, Zhong J, Debuc DC, Tao A, Xu Z, Lam BL (2014). Functional slit lamp biomicroscopy for imaging bulbar conjunctival microvasculature in contact lens wearers. Microvasc Res.

[22] Deng Z, Wang J, Jiang H, Fadli Z, Liu C, Tan J (2016). Lid wiper microvascular responses as an indicator of contact lens discomfort. Am J Ophthalmol.

[23] Shi Y, Hu L, Chen W, Qu D, Jiang H, Wang J. Evaluated conjunctival blood flow velocity in daily contact lens wearers. Eye Contact Lens. 2018;44(Suppl1):S238–43. 10.1097/ICL.0000000000000389 PMC5640452 28410281

[24] Chen W, Batawi HI, Alava JR, Galor A, Yuan J, Sarantopoulos CD (2017). Bulbar conjunctival microvascular responses in dry eye. Ocul Surf.

[25] Chen W, Deng Y, Jiang H, Wang J, Zhong J, Li S (2018). Microvascular abnormalities in dry eye patients. Microvasc Res.

[26] Deneux T, Takerkart S, Grinvald A, Masson GS, Vanzetta I (2012). A processing work-flow for measuring erythrocytes velocity in extended vascular networks from wide field high-resolution optical imaging data. Neuroimage..

[27] Koutsiaris AG, Tachmitzi SV, Batis N, Kotoula MG, Karabatsas CH, Tsironi E, et al. Volume flow and wall shear stress quantification in the human conjunctival capillaries and post-capillary venules *in vivo*. Biorheology. 2007;44(5–6):375–86. 18401076

[28] Tălu S (2011). Fractal analysis of normal retinal vascular network. Oftalmologia..

[29] Doubal FN, MacGillivray TJ, Patton N, Dhillon B, Dennis MS, Wardlaw JM (2010). Fractal analysis of retinal vessels suggests that a distinct vasculopathy causes lacunar stroke. Neurology..

[30] Cheung N, Donaghue KC, Liew G, Rogers SL, Wang JJ, Lim SW (2009). Quantitative assessment of early diabetic retinopathy using fractal analysis. Diabetes Care.

[31] Schulze MM, Hutchings N, Simpson TL (2008). The use of fractal analysis and photometry to estimate the accuracy of bulbar redness grading scales. Invest Ophthalmol Vis Sci.

[32] Chen W, Xu Z, Jiang H, Zhou J, Wang L, Wang J (2017). Altered bulbar conjunctival microcirculation in response to contact lens wear. Eye Contact Lens..

[33] Hu L, Shi C, Jiang H, Shi Y, Sethi Z, Wang J (2018). Factors affecting microvascular responses in the bulbar conjunctiva in habitual contact lens wearers. Invest Ophthalmol Vis Sci.

[34] Morgan PB, Efron N, Brennan NA, Hill EA, Raynor MK, Tullo AB (2005). Risk factors for the development of corneal infiltrative events associated with contact lens wear. Invest Ophthalmol Vis Sci.

[35] Efron N, Morgan PB (2006). Rethinking contact lens associated keratitis. Clin Exp Optom.

[36] Morgan P B (2005). Incidence of keratitis of varying severity among contact lens wearers. British Journal of Ophthalmology.

[37] Alzahrani Y, Colorado L, Pritchard N, Efron N (2016). Inflammatory cell upregulation of the lid wiper in contact lens dry eye. Optom Vis Sci.

[38] Alzahrani Y, Colorado LH, Pritchard N, Efron N (2017). Longitudinal changes in Langerhans cell density of the cornea and conjunctiva in contact lens-induced dry eye. Clin Exp Optom..

[39] Stern ME, Gao J, Siemasko KF, Beuerman RW, Pflugfelder SC (2004). The role of the lacrimal functional unit in the pathophysiology of dry eye. Exp Eye Res.

[40] Pouyeh Bozorgmehr, Viteri Eduardo, Feuer William, Lee David J., Florez Hermes, Fabian James A., Perez Victor L., Galor Anat (2012). Impact of Ocular Surface Symptoms on Quality of Life in a United States Veterans Affairs Population. American Journal of Ophthalmology.

